# Intelligent biofilm detection with ensemble of deep learning networks

**DOI:** 10.4317/medoral.26937

**Published:** 2025-02-15

**Authors:** Brenda Pereira Pinheiro Sobrinho, Bernardo Peters Menezes Silva, Katia Montanha de Andrade, Bruna Pereira Pinheiro Sobrinho, Daniel Araki Ribeiro, Jean Nunes dos Santos, Luciano Rebouças de Oliveira, Patricia Ramos Cury

**Affiliations:** 1 PosGraduate Program in Dentistry and Health, School of Dentistry, Federal University of Bahia, Salvador, Brazil; 2 Intelligent Vision Research Lab, Institute of Computing, Federal University of Bahia, Salvador, Brazil; 3 Undergraduate Program in Dentistry and Health, School of Dentistry, Federal University of Bahia, Salvador, Brazil; 4 Department of Biosciences, Institute of Health and Society, Federal University of São Paulo, UNIFESP, Santos, SP, Brazil

## Abstract

**Background:**

Dental biofilm is traditionally identified visually, which can be challenging and time-consuming due to its color similarity with the tooth. The aim of this study was to evaluate the performance of U-Net neural networks for the automatic detection of dental biofilm without disclosing agents on intraoral photographs of deciduous and permanent teeth using an ensemble strategy.

**Material and Methods:**

This retrospective exploratory study was conducted on two datasets of intraoral images obtained from deciduous and permanent dentitions. The first dataset was used to validate dental biofilm annotations by an expert with disclosing agents. The second dataset, without disclosing agents, was employed to train and evaluate the U-Net neural network in the identification of dental biofilms using an ensemble strategy.

**Results:**

The performance of the ensemble method was assessed using a cross-validation procedure, with six groups dedicated to training, one group for validation, and one group exclusively taken as a test set for the final evaluation of the ensemble. The performance of the neural network was evaluated using accuracy, F1 score, sensitivity, and specificity. The U-Net neural network achieved an accuracy of 93.1%, sensitivity of 65.1%, specificity of 95.9%, and an F1 score of 63.0%.

**Conclusions:**

The U-Net neural network using the ensemble strategy was able to automatically identify visually detecTable dental biofilms on intraoral photographs. The application of this new knowledge will soon be available in clinical practice.

** Key words:**Artificial intelligence, dental biofilm, ensemble method, photography, preventive dentistry.

## Introduction

Dental surfaces are exposed to colonization by different microorganisms that form the dental biofilm, the main etiological factor of oral diseases such as dental caries, gingivitis, and periodontitis. However, detecting dental biofilm is difficult for both the patient and the dentist or hygienist because of its similar color to the tooth surface (1). Dental biofilms can be identified using a disclosing solution. Such solutions use dyes that modify the color of the biofilm so that it contrasts with the surface of the tooth (2). However, this procedure has disadvantages, such as the temporary staining of mucous membranes and restorations (3-5), as well as the unpleasant taste of the disclosing agent. Thus, further investigation is needed to develop a convenient method for the detection and quantification of dental biofilms.

Artificial intelligence (AI) has gained visibility in dentistry in recent years because it can be used to replicate dentists actions to perform tasks automatically (6). Machine learning (ML) is a sub-area of AI that uses algorithms to learn from data, adjusting its own internal parameters to automatically improve performance in specific tasks (7). Deep learning is a ML technique designed to automate tasks that focuses on the creation and training of deep neural network algorithms for complex data processing tasks and high-level pattern recognition and that can generate information such as image classification, detection, and segmentation (8). In dentistry, examples that highlight the application of deep learning are its use for automatic dental biofilm detection on intraoral photographs (3,9,10). However, the model’s performance is related to the availability of large datasets for training the neural network, which can limit the effectiveness of the technique (10).

The ensemble strategy is used in AI to deal with challenges such as small sample sizes and irregular and discrepant distribution of classes and data (11). The neural network receives a labeled database as input and generates masks as output. Using the ensemble strategy, the average output score is calculated by the combination of multiple algorithms to produce more reliable and accurate results (12). By using the generated model, cohesive predictions can be made for new unlabeled examples (13). In short, several individual models are trained independently and their predictions are then combined to generate a final prediction. The aim of the present study was to assess the performance of U-Net neural networks in the automatic detection of dental biofilm without disclosing agents on intraoral photographs of deciduous and permanent teeth using an ensemble strategy.

## Material and Methods

Study design and data collection

The present study is a retrospective exploratory study in which two databases of randomly chosen intraoral photographs were obtained from a private clinic in Salvador, Bahia, between 10 September 2019 and 6 March 2023. The project was approved by the Research Ethics Committee of the School of Dentistry, Federal University of Bahia (protocol number: 4.434.730), and was conducted in accordance with the Declaration of Helsinki. This study is described following the Checklist for Artificial Intelligence in Medical Imaging.

The initial dataset consisted of 96 intraoral photographs from 16 participants captured before and after the application of a dental biofilm disclosing agent (Eviplac Tablets, Biodinamicas™). This dataset was used to assess the intraexaminer agreement in biofilm detection. Consistency of the examiner annotations was showed previously (10). The examiner’s annotation was tested using the intraclass correlation coefficient (ICC), comparing the dental biofilm area on each tooth based on digital images without and with the disclosing agent. The ICC was 0.93 (95% CI, 0.92-0.94). The dental biofilm labeled by the dentist (without disclosing agent) was thus defined as the ground truth.

**Figure 1:**
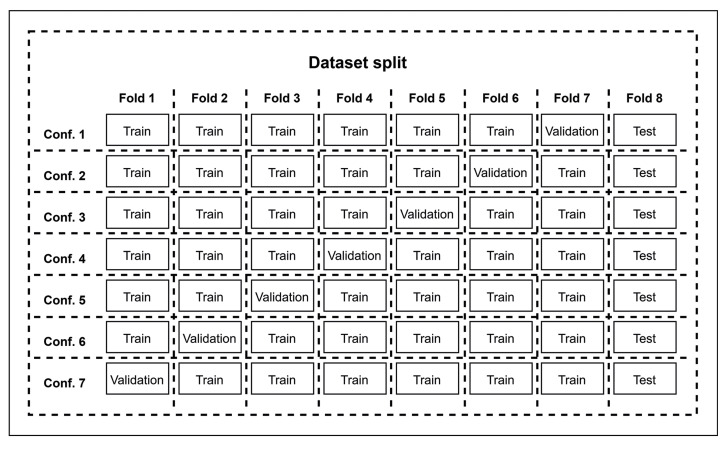
Splitting of the dataset into eight folds for the training, validation, and test sets.

The second dataset, the experimental one, was composed of 480 intraoral images obtained without disclosing agents from 160 people with or without orthodontic appliances. This dataset was used to train and evaluate a neural network for segmentation of the dental biofilm. The database was randomly subdivided into eight groups: training (360 images), validation (60 images), and testing (60 images). The training and validation groups alternated, with each group taking turns being used for training and validation in a cross-validation process. The eighth group was used as a test subset with 60 images selected for the ensemble strategy (Fig. 1).

Eligibility criteria of the images were the absence of clinically visible caries or tetracycline stain, high-resolution and focused images, and frontal and lateral photographs (left and right) taken in the perpendicular plane in buccal views. Low-light images and images containing saliva bubbles or blurred areas were excluded from this study. Furthermore, the patient identification was removed from all captured images and each participant was identified by randomly assigning letters.

During image acquisition, each participant used a plastic lip retractor. The intraoral photographs were obtained with a Canon EOS Rebel T3 camera (4272 x 2848 pixels; approximately 3 Megabytes), a Canon Macro 100 lens, and a Canon MR-14 flash ring and were stored in JPEG format. The default camera settings were aperture f/29, ISO 200, shutter speed 1/160, and automatic white balance. The flash was set to ETTL mode.

- Biofilm annotation of the experimental dataset

Yellowish areas, loss of shine, and a rough or granular appearance were considered dental biofilm. The annotions were made using frontal view photographs to segment the dental biofilm of incisors and lateral view photographs for canines, premolars, and first molars.Two annotations were created for the experimental dataset using the polygon tool of the COCO Annotator 0.11.1 open-source image segmentation software. The first annotation consisted of marking the dental biofilm on all teeth of the image, which provided binary masks to separate the biofilm into background pixels. These binary masks were used in the training and validation procedures. The second annotation consisted of tooth instance segmentation, with 60 images being used exclusively for evaluation of the neural network and for statistical analysis. The buccal surface of each tooth was defined as the region of interest.

- Cross-validation

In the cross-validation procedure, the dataset was partitioned into eight distinct subsets, each consisting of 60 images from 20 patients (Fig. 1). During each iteration, six of these subsets were allocated to the model’s training phase (360 images), while one subset was assigned to the validation phase (60 images), alternating between groups in each subsequent iteration. This procedure ensures that each subset acts as both a training group and a validation group. During the iterations, performance metrics such as sensitivity, specificity, accuracy, and F1 score were recorded, which provide a comprehensive view of the model’s behavior throughout training and validation.

The eighth subset was defined exclusively as the test group (60 images), which remained unchanged during the cross-validation iterations. This approach was adopted in order to later use this last group as a test set for evaluation of the model’s performance using the ensemble strategy.

- Ensemble strategy

Each base model, trained in the cross-validation iterations, generated its own prediction. These preditions were then combined to form a final prediction. After completion of the iterative process and analysis of performance of the models, the eighth group was strategically kept aside to serve as an independent and unbiased test set, enabling rigorous and unbiased assessment of the ensemble model performance.

- Model

**Figure 2:**
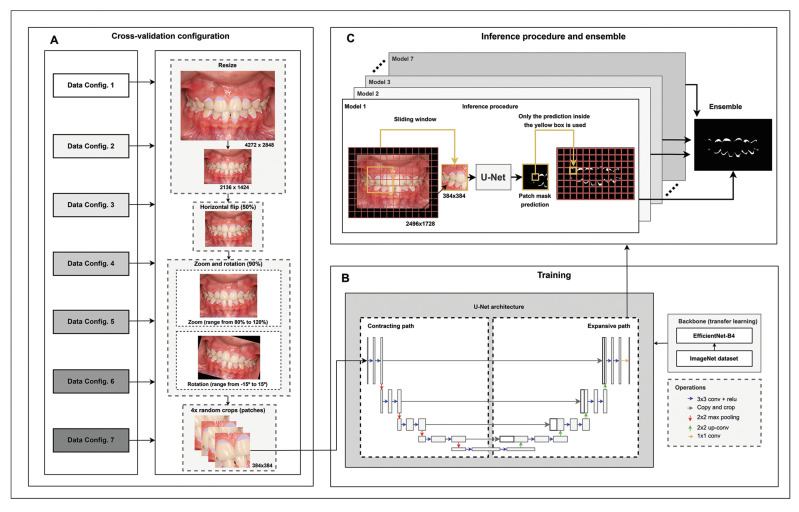
Pre-processing, data augmentation, training, and evaluation procedures repeated seven times in a cross-validation manner. A: Pre-processing and data augmentation - The photographs and labels of the experimental dataset were resized, and data augmentations were applied during each step. At the end of each step, 4 random patches were selected. B: Training - The training procedure employed supervised learning, in which the model outputs were compared with the ground truth to adjust the Convolutional Neural Network. Transfer learning was applied using an EfficientNet B4 trained on the ImageNet dataset chosen as the backbone of the U-Net. C: The model’s inference procedure used a sliding-window approach employing only each window center.

The RGB image was taken as input and the binary masks as output. Instead of entering the full high-resolution images into the model, fixed-size patches of 384 x 384 pixels were used. The patch size was chosen based on preliminary tests following the original inputs from the EfficientNet encoders. A U-Net architecture was used for image segmentation (Fig. 2), employing a publicly available implementation written in the PyTorch framework (10). Furthermore, an EfficientNet-B4 trained on the ImageNet (transfer learning) dataset was chosen as the base structure of the U-Net architecture. The EfficientNet-B4 architecture was selected because this conFiguration showed the best results (10).

- Pre-processing and data augmentation

All images were pre-processed by reducing their width and height by 50%, resulting in a resolution of 2136 x 1424 (Fig. 2). During training, the data were artificially augmented. Fig. 2 illustrates the data augmentation procedure: (i) a horizontal flip 50% of the time, and (ii) a random zoom from 80% to 120% and a random rotation from -15 to 15 degrees at 90% of the time. After each augmentation step, four random 384 x 384 patches from each image were selected to train the U-Nets in a supervised manner (Fig. 2).

- Data training

The training procedure was run without time concerns on 8 Tesla V100 16GB GPUs using a supervised learning approach. The validation procedure and final evaluation of the model (Fig. 2) were carried out at the end of each epoch using the inference method: each image was filled with black pixels around the edges until a size of 2496 x 1728 pixels was reached. Predictions on 384 x 384 patches were computed with 96-pixel steps using the sliding-window approach. Only the central 96 x 96 square of the network predictions was used. The confidence score ranged from 0 to 1, with 0 corresponding to the lowest confidence level and 1 to the highest level, using a sigmoid function (10). For dichotomous categorization, dental biofilm detection was defined when the confidence scores produced by the network exceeded the midpoint of 0.5 (14). The model that obtained the highest F1 score for the validation dataset was maintained. The training procedure was repeated for all combinations of cross-validation groups, resulting in seven trained models to be later combined in the ensemble strategy.

- Evaluation of the U-net neural networks

Quantitative and qualitative analyses were applied to evaluate whether the proposed deep learning solutions could correctly segment dental biofilms. First, quantitative analysis was performed using dichotomous categorization in which the dental biofilm detected by the model was compared with the ground truth labeled by the dentist. For the ensemble strategy, the output scores of all seven models were averaged to obtain the final scores. Next, qualitative analyses of the most and least satisfactory network results per patient were carried out, comparing the binary ground-truth masks with the dichotomous predictions and the output confidence scores.

- Statistical analysis

The results of the deep learning model were compared to the ground truth, providing counts of true positives, true negatives, false positives, and false negatives for each pixel. The following metrics were calculated per tooth: accuracy, sensitivity, specificity, and F1 score. Accuracy was calculated as the proportion of true positives and negatives in all cases evaluated. The statistics were computed using Python and the SciPy 3.7.7 package.

Results  

A total of 480 intraoral images from 160 participants aged 5 to 73 years with all teeth present and without caries or periodontal diseases were analyzed. In the initial dataset, 3 images from one participant were excluded because they were out of focus.

- Quantitative analysis

Table 1 shows the mean metrics according to the presence of orthodontic appliances and image type (front or lateral view). The performance of the ensemble strategy was adequate, with a mean accuracy of 93.1%, sensitivity of 65.1%, specificity of 95.9%, and F1 score of 63%.

- Qualitative analysis

The worst results were obtained with images that contained a lower amount of dental biofilm. Otherwise, the best results were obtained with images with more dental biofilm.

## Discussion

The present study evaluated U-Net neural networks using an ensemble strategy to identify dental biofilms on intraoral photographs of deciduous and permanent teeth of individuals with or without orthodontic appliances. Analysis of this neural network showed a satisfactory performance, with a mean accuracy of 93.1%, sensitivity of 65.1%, specificity of 95.9%, and F1 score of 63.0%. These results indicate that the trained network employing the ensemble strategy can be used for the automatic identification of dental biofilm on intraoral digital images.

Studies have highlighted different approaches and obstacles in dental biofilm identification that range from specific methodological limitations to restrictions related to sample size, emphasizing the diverse challenges faced by research in this field. Compared to the U-Net model of a previous study that did not use the ensemble method and achieved an accuracy of 91.8% (10), sensitivity of 67.2%, specificity of 94.4% and F1 score of 60.6%, the present study applying the ensemble strategy obtained better network performance. One survey on dental biofilm identification without the use of the ensemble method was restricted to a single primary tooth per intraoral image (3). Other studies used small datasets consisting of intraoral photographs from only 25 and 20 participants (9,15). In contrast, the present study included 480 intraoral images from individuals aged 5 to 73 years with primary and permanent teeth, providing the network with a more extensive set of comprehensive, diverse, and valuable data.

Accurate and comprehensive annotation by accurately delineating the dental biofilm not only provides the essential basis for training the base models but also significantly affects the robustness and reliability of the ensemble strategy. However, the characteristics of the dental biofilm such as a color similar to the tooth and poorly defined and irregular contours compromised the segmentation, labeling and classification of the biofilm, resulting in relatively low F1 scores. To overcome these limitations, the expert annotated the second database using the polygon tool of COCO Annotator, clicking on the limits of the dental biofilm area. This approach reduced interactions and allowed for more accurate segmentation compared to having to paint the entire biofilm area with a brush tool (10).

Qualitative analysis, the neural network revealed more satisfactory performance for intraoral images with more significant dental biofilm, in agreement with a previous study (10). Performance was superior in the cervical third of the teeth, especially in canines, premolars, and first molars, and in cases with braces and orthodontic wires because of greater biofilm accumulation (13,16). This fact highlights the continued need for persistence of the challenge improving the ability of neural networks to effectively deal with situations of less dental biofilm accumulation.

One limitation of this study, with implications for its reproducibility, is that only photographs taken by a professional camera were included. In addition, only one expert annotated the dental biofilm. It would be important that more professionals participate in the annotation process to mitigate the risk of bias. Another limitation was the exclusion of second and third molars from this analysis because of the frequent difficulty in obtaining adequate visualization of these teeth on photographs. The same applies to the palatal, lingual, and occlusal surfaces of all teeth in the oral cavity. One study used a scanner, a device capable of capturing all sides of the teeth, to label the biofilm throughout the dental arch (15). However, the image quality was compromised, showing pixelation and blurring especially in the proximal areas, which can impair visual detection of dental biofilm.

Developing a tool for dental biofilm identification can have a significant impact on the prevention, treatment and maintenance of oral health, benefiting both patients and professionals in clinical practice. A tool capable of assisting in dental biofilm detection may guide the patient in order to improve oral hygiene based on immediate feedback about areas where brushing and flossing could be improved. In addition, it would be possible for the professional to monitor the patient’s progress throughout treatment, ensuring that biofilm self-control is adequate. Within this context, the results of the present study highlight the feasibility of biofilm identification using deep learning techniques, paving the way for the development of a platform that carries out this recognition in an automated manner.

A broader database is recommended for future research to improve the performance of the neural network. This can be achieved by incorporating more images from different devices, including cell phone and Tablet cameras. These images must be captured under varying lighting conditions. Furthermore, the annotation task should be performed by multiple examiners. Considering that specific oral conditions can affect dental biofilm distribution, the database of future projects should include patients with fixed and implant-mounted partial dentures, different crowding conditions, distinct patterns of malocclusion, cases of carious lesions, variable roughness of the enamel and dentin surfaces, and teeth with varied colors. These factors would render the dataset more diverse and thus allow more effective neural network training, improving its biofilm detection ability.

In conclusion, the ensemble method of U-Net neural networks was able to automatically identify visually detecTable dental biofilms on intraoral photographs; it is therefore an automated, objective, and quantitative option for dental biofilm detection. As more data are incorporated into the neural network for labeling and segmentation training, there will be significant potential for improvement of the network.

## Figures and Tables

**Table 1:** Performance metrics (accuracy, sensitivity, specificity and F1 score) of the ensemble model for the entire dataset and subgroups (with and without orthodontic appliances or frontal and lateral views).

Image type	Accuracy (%)	Sensitivity (%)	Specificity (%)	F1 score (%)
No orthodontic appliance	92.4	71.7	94.5	63.1
Orthodontic appliance	93.7	58.8	97.2	62.9
Frontal view	91.9	67.9	94.0	57.3
Lateral view	92.6	73.3	94.7	65.8
Total	93.1	65.1	95.9	63.0
